# Use of K-Y Jelly on Throat Packs for Postoperative Sore Throat after Nasal Surgery: A Randomized Controlled Trial

**DOI:** 10.1055/s-0043-1776724

**Published:** 2024-01-04

**Authors:** Ahmed Mahmoud M.M. Elgarhy, Saeed Mostafa Abdelhameed, Othman Saadeldien Yahia, Wael Mohamed Elmahdy Ibrahim, Tamer Mohamed Ahmed Ewieda, Mahmoud M. Elsayed, Marwa M. Abdel-aziz, Naglaa A. Elshehawy, Hussein Magdy Abdelkader, Mahmoud Hamdy Al Boghdady, Ayman Yehia Abbas

**Affiliations:** 1 Department of Intensive Care and Pain Management, Division of Anesthesia, Al-Azhar University, Cairo, Egypt; 2 Department of Otorhinolaryngology, Al-Azhar University-Assuit Branch, Al-Azhar University, Assuit, Egypt

**Keywords:** nasal surgery, postoperative sore throat, K-Y jelly, throat packs

## Abstract

**Introduction** Postoperative sore throat (POST) is a fairly common side effect of general anesthesia. The K-Y jelly is a well-known lubricant used in many medical procedures.

**Objective** In this randomized study, we evaluated the use of throat packs soaked with K-Y jelly for POST outcomes in patients submitted to nasal surgery.

**Methods** The present double-blinded, randomized, controlled study included 140 ASA I–II patients undergoing nasal surgery under general anesthesia. Patients received either or K-Y jelly or water-soaked X-ray detectable throat packs fully inserted into the mouth to occlude the oropharynx.

**Results** Comparison between the studied groups regarding the severity of POST assessed by visual analog scale revealed significantly lower POST levels in the K-Y jelly group on recovery from anesthesia, and at 2, 4, and 6 hours postoperatively.

**Conclusions** The use of K-Y jelly-soaked throat packs was associated with less severe POST after nasal surgery.

## Introduction

Postoperative sore throat (POST) is a well-documented adverse effect following general anesthesia. The condition usually results from nerve compression and mucosal injury related to the anesthetic procedures. 1 Associated risk factors include younger age, female sex, and prolonged anesthesia. 2 Patients subjected to nasal surgery have additional iatrogenic cause of POST. In those patients, water-soaked nasopharyngeal packs are used to reduce the risk of postoperative nausea and vomiting (PONV). 3 However, use of these packs is associated with significant POST. 4 5 In spite of the fact that POST is a self-limiting condition, most patients identify it as one of the most disturbing postoperative symptoms. 6

Suggested techniques for reduction of this problem include administration of topical and systemic lidocaine, 7 topical benzydamine hydrochloride, 8 intravenous dexamethasone, 9 topical ketamine, 10 topical corticosteroids, 11 aerosolized corticosteroids, 12 topical licorice, 13 topical magnesium, 14 and intravenous dexmedetomidine 15 with a variable spectrum of efficacy and side effects.

Notably, most trials managed to use miscellaneous biochemical agents with different mechanisms to reduce the sequences of traumatic injury responsible for the sore throat. In this study, we try to assess the role of other agents that exert their actions mainly through mechanical effects.

The K-Y jelly is a well-known lubricant used in many medical procedures, including bronchoscopy, esophagoscopy, 16 gonioscopy, 17 and cryotherapy for odontogenic keratocysts. 18 In a randomized clinical study, K-Y lubrication of the tracheal tubes was found to be superior to lidocaine jelly in prevention of postoperative sore throat. 19 Interestingly, one recent experimental study highlighted the value of this brand of jelly in inhibition of the increase in cuff pressure during general anesthesia. 20

In this randomized study, we evaluated the role of throat packs soaked with K-Y jelly for POST and PONV after nasal surgery.

## Patients and Methods

### Setting, Design and Ethical Considerations

The present study is a multi-centric double-blinded randomized controlled trial. The study protocol was approved by the local ethical committee. The study is registered at clinicaltrials.gov (NCT05436743).

The study included patients under the American Society of Anesthesiologists (ASA) classification from I to II, all of whom were undergoing nasal surgery under general anesthesia. Patients were excluded from the study if they had gastroesophageal reflux, regurgitation, history of postoperative sore throat, nasal surgery for malignant disease, concurrent or recent use of systemic or topical agents for sore throat, or airway Mallampati grade > 2.

### Sample Size Calculation

In the metanalysis of Wang et al., 21 the authors suggested glycyrrhiza (licorice) as the most successful topical agent for reduction of postoperative sore throat. In another metanalysis involving only RCTs, including licorice, the pooled number of sore postoperative throat events in the licorice group was 71/324 (21.9%) versus 177,250 (46.8%) in the control group. 13 Using this data in the G Power (Kiel University, Germany) software, version 3.1.9.6, at an α error probability of 5%, a study power of 80%, and an allocation ratio of 1, the estimated sample size was of 63 patients for each group. To avoid bias related to loss of follow-up, we added 10% of patients to each study group. As such, the final estimated number for each group is of 70 patients.

### Randomization and Blinding

All patients included in the present study were equally and randomly allocated to one of the study groups using simple randomization by computer-generated tables. Both patients and outcome assessors were blinded to allocation groups. The sealed envelope technique was used for this purpose. Randomization and blinding were secured by an independent researcher who wasn't aware of the study's nature and design.

### Anesthetic Procedure

Patients were premedicated with oral lorazepam 0.04 mg/kg the night before surgery. A throat pack detectable by X-ray was soaked with either water or K-Y jelly and fully inserted into the mouth to occlude the oropharynx. Induction of anesthesia was achieved using fentanyl 1 to 2 µg/kg and propofol 2 mg/kg. Furthermore, vecuronium bromide 0.1 mg/kg was used to facilitate tracheal intubation.

A Macintosh laryngoscope blade was used to perform direct laryngoscopy. The anesthetist who performed both endotracheal intubation and laryngoscopy was also blinded to the study group allocations. Lung ventilation was achieved using FiO2 0.4, maintaining the end-tidal CO _2_ between 32 and 35 mmHg. Anesthesia was maintained with inhalational isoflurane at 1.2% minimum alveolar concentration (MAC). A combination of glycopyrolate 0.01 mg/kg and neostigmine 0.05 mg/kg was used to reverse neuromuscular blockade at the end of surgery, and patients were extubated and moved to the post-surgery care unit.

### Outcome Assessment

The primary end point was POST severity. Secondary end point was occurrence of other side effects. Severity of POST was assessed using a visual analog scale (VAS, from 0 to 100; where 0 means no sore throat and 100 means worst imaginable sore throat). Furthermore, POST and side effects were assessed at recovery from anesthesia, then at 2, 4, and 6 hours postoperatively, both at rest and on swallowing.

### Statistical Analysis

Data obtained from the present study were expressed as number (N) and percentages (%) or as mean and standard deviation (SD). Categorical variables were compared using the chi-square test while numerical variables were compared using the t-test. All statistical procedures were executed using the Statistical Package Social Sciences (SPSS, IBM Corp., Armonk, NY, USA), version 25.0, with *p* -values less than 0.05 being considered statistically significant.

## Results

The present study included 140 patients submitted to nasal surgery. They were randomly and equally allocated to one interventional group. Patients in both groups were comparable regarding age, sex distribution, body mass index (BMI), ASA classification, type of surgery, and duration of surgery ( Table 1 ). Comparison between the studied groups regarding the severity of POST postoperatively assessed by VAS revealed significantly lower levels in the K-Y jelly group on recovery from anesthesia, when compared with the control group (resting: 23.6 ± 5.2 vs. 28.7 ± 7.7, *p* = 0.002; swallowing: 31.0 ± 5.3 vs. 35.9 ± 7.3, *p* = 0.002), 2 hours (resting: 16.1 ± 6.0 vs. 21.7 ± 8.9, *p* = 0.005; swallowing: 23.6 ± 5.0 vs. 27.7 ± 7.8, *p* = 0.011), 4 hours (resting: 11.9 ± 2.7 vs. 15.4 ± 6.9, *p* = 0.007; swallowing: 16.0 ± 4.8 vs. 20.3 ± 7.9, *p* = 0.008), and 6 hours (swallowing: 12.6 ± 3.3 vs. 16.0 ± 6.6, *p* = 0.008).

**Table 1:** Baseline data in the studied groups

	K-Y jelly group N = 70	Control group N = 70	*p* -value
**Age (years)** , mean ± SD	32.3 ± 7.6	30.0 ± 5.2	0.13
**Male/female*,*** n	34/36	38/32	0.63
**BMI** (Kg/m ^2^ )	25.6 ± 2.6	24.7 ± 1.4	0.11
**ASAI/II** , n	40/30	48/22	0.32
**Surgery** , n (%)			
FESS	42 (60.0)	50 (71.4)	0.31
Rhino	28 (40.0)	70 (28.6)	
**Duration of surgery** (min.), mean ± SD	146.0 ± 19.0	151.7 ± 23.2	0.26

**Abbreviations:** ASA, American Society of Anesthesiology; BMI, body mass index; FESS, functional endoscopic sinus surgery, SD, standard deviation.

**Fig. 1:**
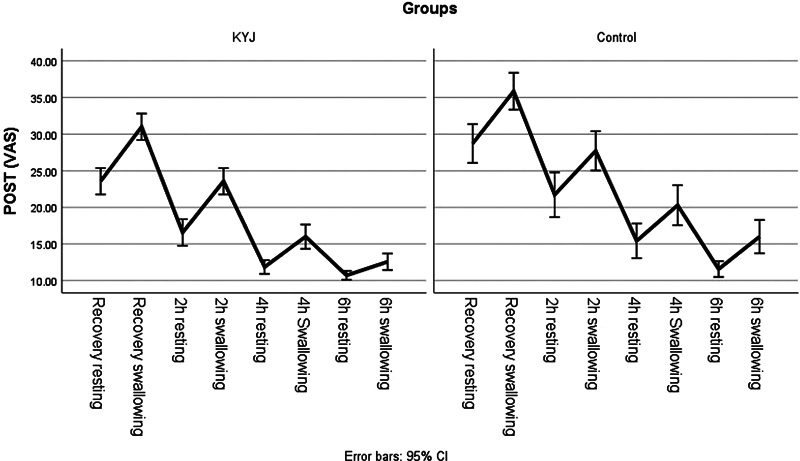
POST scores in the studied groups.

No significant differences were found between POST levels at rest, 6 hours postoperatively (10.7 ± 1.8 vs. 11.6 ± 3.2, *p* = 0.17) ( Table 2 , Fig. 1 ). Furthermore, PONV was encountered in 6 (8.6%) patients in the K-Y jelly group, and in 2 (2.9%) of the control group, with no significant differences ( *p* = 0.3) ( Table 2 , Fig. 1 ).

**Table 2:** Outcome parameters in the studied groups

	K-Y jelly group N = 70	Control group N = 70	*p* -value
**POST** (VAS), mean ± SD			
Recovery			
- Resting	23.6 ± 5.2	28.7 ± 7.7	0.002
- Swallowing	31.0 ± 5.3	35.9 ± 7.3	0.002
2 hours postoperatively			
- Resting	16.1 ± 6.0	21.7 ± 8.9	0.005
- Swallowing	23.6 ± 5.0	27.7 ± 7.8	0.011
4 hours postoperatively			
- Resting	11.9 ± 2.7	15.4 ± 6.9	0.007
- Swallowing	16.0 ± 4.8	20.3 ± 7.9	0.008
6 hours postoperatively			
- Resting	10.7 ± 1.8	11.6 ± 3.2	0.17
- Swallowing	12.6 ± 3.3	16.0 ± 6.6	0.008
**PONV** , n (%)	6 (8.6)	2 (2.9)	0.3

**Abbreviations:** PONV, postoperative nausea and vomiting; POST, postoperative sore throat; SD, standard deviation; VAS, visual analog scale.

## Discussion

The present randomized controlled study revealed significantly lower POST severity in patients with the K-Y jelly-soaked X-ray detectable throat packs, in comparison to those with the water-soaked ones. Moreover, we couldn't find significant differences between the studied groups regarding the prevalence of PONV.

The use of throat packs as prophylaxis against PONV and POST was previously assessed in a randomized clinical study. 5 The study concluded that throat packs don't provide beneficial effects on PONV rates, nor on POST severity. Moreover, the authors noted that throat packs were associated with more severe POST in the initial recovery period after nasal surgery.

The present study revealed that lubrication of the throat packs with K-Y jelly efficiently reduced pain in the early postoperative period. This brand jelly was successfully used to reduce POST in other trials without throat packs. When the tracheal tubes were lubricated with K-Y jelly before insertion, the severity of POST was significantly lower than with a water-soluble 1% hydrocortisone cream. 22

In comparison with other biochemical agents, K-Y jelly has a well-known safety profile. Its use doesn't interfere with postoperative recovery of patient's reflexes and there are no known interactions with anesthetic drugs or other medications.

## Conclusion

In conclusion, the present study found that use of throat packs soaked with K-Y jelly is associated with less severe POST. The findings of the present study are limited due to its single-center nature. Additionally, this study included multiple nasal surgeries that can affect outcome assessment.
